# Mosquitoes: Important Sources of Allergens in the Tropics

**DOI:** 10.3389/falgy.2021.690406

**Published:** 2021-07-08

**Authors:** Jose Fernando Cantillo, Leonardo Puerta

**Affiliations:** ^1^Institute for Immunological Research, University of Cartagena, Cartagena, Colombia; ^2^Inmunotek, S.L., Madrid, Spain

**Keywords:** mosquito allergy, allergens, tropics, IgE, *Aedes aegypti*, cross reactivity

## Abstract

There are more than 3,000 mosquito species. *Aedes aegypti, Ae. communis*, and *C. quinquefasciatus* are, among others, three of the most important mosquito allergen sources in the tropics, western, and industrialized countries. Several individuals are sensitized to mosquito allergens, but the epidemiological data indicates that the frequency of sensitization markedly differs depending on the geographical region. Additionally, the geographical localization of mosquito species has been affected by global warming and some mosquito species have invaded areas where they were not previously found, at the same time as other species have been displaced. This phenomenon has repercussions in the pathogenesis and the accuracy of the diagnosis of mosquito allergy. Allergic individuals are sensitized to mosquito allergens from two origins: saliva and body allergens. Exposure to saliva allergens occurs during mosquito bite and induces cutaneous allergic reactions. Experimental and clinical data suggest that body allergens mediate different manifestations of allergic reactions such as asthma and rhinitis. The most studied mosquito species is *Ae. aegypti*, from which four and five allergens of the saliva and body, respectively, have been reported. Many characterized allergens are homologs to arthropod-derived allergens, which cause strong cross-reactivity at the humoral and cellular level. The generalized use of whole body *Ae. communis* or *C. quinquefasciatus* extracts complicates the diagnosis of mosquito allergy because they have low concentration of saliva allergens and may result in poor diagnosis of the affected population when other species are the primary sensitizer. This review article discusses the current knowledge about mosquito allergy, allergens, cross-reactivity, and proposals of component resolved approaches based on mixtures of purified recombinant allergens to replace saliva-based or whole-body extracts, in order to perform an accurate diagnosis of allergy induced by mosquito allergen exposure.

## Introduction

Mosquitoes are insects that belong to the family Culicidae, which includes more than 3,000 species distributed worldwide. Some species have the ability to adapt to different climatic conditions. Four species, *Culex pipiens, Culex quinquefasciatus, Aedes aegypti*, and the genera Anopheles have virtually populated all the planet and induce allergic reactions in atopic individuals (1).

Mosquito allergy occurs worldwide and is common in tropical and subtropical regions where mosquitoes are abundant, since the climatic conditions at these latitudes favor their life cycle and proliferation (2, 3), and increase the chances of interaction with humans. Early efforts to identify mosquito allergens focused mainly on the saliva because it was believed that biting was the unique mechanism of exposure and sensitization. However, some evidence suggests that proteins from the insect's body may remain in the environment as aerosols or in the dust after they die and induce and allergic responses when they are inhaled by atopic individuals, similarly as house dust mites (HDMs) do.

Mosquito allergy seems to be highly prevalent and variable, although there is not enough data to support such affirmation. Diagnosis criteria is different, dependent of the study design or clinicians team. In some studies, the diagnosis of mosquito allergy was defined by bite reactions or in severe cases, anaphylaxis and systemic symptoms after a witnessed mosquito bite. Diagnosis was also made in some cases by SPT to mosquito allergen extract or positive serum to mosquito saliva IgE (4). In Monterrey City, Mexico, a cross-sectional study reported that 82% of patients admitted to the allergy service had specific IgE to mosquitoes, although only 2.5% of them showed positive skin reactions (5). In a study performed in India, 47% of the population with asthma and/or allergic rhinitis were sensitized to mosquito allergens, as determined by skin prick tests (SPT), serum specific IgE antibodies and bronchial provocation tests with whole mosquito body extracts (6). In Guangzhou, China, a study showed that in a cohort of 7,047 allergic patients, 4% of them had detectable specific IgE levels to mosquito allergens, ranging from ≥0.35 to <3.5 IU/ml in most of the patients, with peaks of sensitization at age between 15 and 18 years (7).

About 20 IgE binding proteins are contained in whole body extracts or the saliva from *Ae. aegypti*, but only 10 have been recognized as allergens in the databases (8, 9). Allergens from the saliva induce cutaneous reactions or a systemic response, that rarely occur (10–13). Body allergens could be contained in emanations and mosquito detritus and, when inhaled, induce variable immune responses (14, 15). A small number of mosquito allergens have been obtained and characterized. More research remains to be performed to establish the complete allergenic spectrum of *Ae. aegypti* and other species.

Studies on the cross-reactivity among different mosquito species, and with other sources of allergens, are scarce. However, an important degree of cross-reactivity between mosquitoes and other arthropods is reported (9, 16). We have found that sera obtained from a cohort of patients residing in the Caribbean island of Martinique suffering from allergic respiratory symptoms after the inhalation of HDM allergens, recognized allergens from *Ae. aegypti* (16). These findings suggest that *Ae. aegypti* contains allergens that induce a Th2 response and subsequent allergic symptoms, or could modulate the response originally established against arthropods.

High occurrence of mosquitoes at patient's homes seems to reflect a higher prevalence of sensitization and may explain a more severe cutaneous reaction during SPTs. In a study performed on a south American population sensitized to cockroaches and mosquitoes, Sanchez et al. (17) found that the size of the wheal generated during SPTs with mosquito extracts is positively correlated with the density of these insects at their homes and directly related with allergy to HDMs. This finding is similar in other tropical countries where high occurrence of mosquitoes and HDMs results in high prevalence of allergic sensitization (18). The observations open questions about the magnitude of the clinical impact produced by sensitization to mosquitoes and postulate the need for developing diagnostic tests to properly identify individuals with mosquito allergy (19). In this context, the comparison of mosquito prevalence and the frequency of sensitization to their allergens in tropical and other regions around the world should be further addressed.

## Mosquito Species: Geographical Distribution and Their Relationship With Allergies

Mosquitoes are arthropods that belong to the class Insecta, order Diptera and members of a family of the nematocerid flies Culicidae. Two subfamilies are widely accepted within the family Culicidae: Anophelinae and Culicinae. Some authors have proposed a third subfamily, Toxorhynchitinae, which includes only one genus (1). Nearly 400 and 2,600 species are included in Anophelinae and Culicinae, respectively. The females of many species of mosquitoes require blood-feeding to reproduce, for which they bite the skin, inject saliva, and then suck blood from vessels (20). Lysozymes, antibacterial glucosidases, anticoagulants, antiplatelet aggregating factors, and vasodilators are molecules contained in mosquito saliva (21–23). Some of these substances induce allergic skin reactions (10–13). We have hypothesized that non-salivary allergens might be contained in emanations and detritus of mosquitoes, and when inhaled, induce respiratory allergic responses (9).

The mosquito species distributed worldwide easily adapt to different environmental conditions helping them to distribute in nearly any latitude (1). Distribution of mosquitoes is generalized to three main geographical locations: Cosmopolitan, Old and New world. In all of these categories, there are species associated with allergic responses. Cosmopolitan: *Anopheles (An.) stephensi, An. minimus, An. sinensis, Ochlerotatus (Oc.) triseriatus, Oc. hendersoni, Culex (Cx.) quinquefasciatus, Cx. tritaeniorhynchus, Cx. pipiens, Cx. pipiens pallens*, and *Cx. tarsalis*. Old world (Africa, Asia, and Europe): *Aedes (Ae.) aegypti, Ae. vexans, Ae. communis, Ae. togoi, Ae. albopictus*, and *Ae. triseriatus*. New world (America): *Culiseta inornate* (Table 1).

**Table 1:** Taxonomical classification and distribution of the main mosquito species associated with mosquito allergy.

**Subfamily Return Tribe**	**Genera**	**Number of subgenera**	**Number of species**	**Distribution**	**Species associated with mosquito allergy**
Anophelinae	*Anopheles*	7	455	Cosmopolitan	*Anopheles (An.) stephensi, An. minimus, An. Sinensis*
	*Bironella*	3	8	Australasian	
	*Chagasia*	-	4	Neotropical	
**Culicinae**					
Aedeomyiini	*Aedeomyia*	2	6	Afrotropical, Australasian, Oriental, Neotropical	
Aedini	*Aedes*	23	363	Old world, Nearctic	*Aedes (Ae.) aegypti, Ae. vexans, Ae. communis, Ae. togoi, Ae. albopictus, Ae. Triseriatus*
	*Argimeres*	2	58	Australasian, Oriental	
	*Ayurakitia*	-	2	Oriental	
	*Borichinda*	-	1	Oriental	
	*Eretmapodites*	-	48	Afrotropical	
	*Haemagogus*	2	28	Principally neotropical	
	*Heizmannia*	2	39	Oriental	
	*Ochlerotatus*	22	550	Cosmopolitan	*Ochlerotatus (Oc.) triseriatus, Oc. Hendersoni*
	*Opifex*	-	1	New Zealand	
	*Psorophora*	3	48	New world	
	*Udaya*	-	3	Oriental	
	*Verrallina*	3	95	Principally Australasian, Oriental	
	*Zeugnomyia*	-	4	Oriental	
Culicini	*Culex*	23	763	Cosmopolitan	*Culex (Cx.) quinquefasciatus, Cx. tritaeniorhynchus, Cx. pipiens, Cx. pipiens pallens, Cx. Tarsalis*
	*Deinocerites*	-	18	Principally neotropical	
	*Galindomyia*	-	1	Neotropical	
	*Lutzia*	3	7	Afrotropical, Australasian, Oriental, Neotropical, Palearctic oriental	
Culisetini	*Culiseta*	7	37	New world, Nearctic	*Culiseta inornata*
Ficalbiini	*Ficalbia*	-	8	Afrotropical, Oriental	
	*Mimomyia*	3	44	Afrotropical, Australasian, Oriental	
Hodgesiini	*Hodgesia*	-	11	Afrotropical, Australasian, Oriental	
Mansoniini	*Coquillettidia*	3	57	Old world, Neotropical	
	*Mansonia*	2	23	Old world, Neotropical	
Orthopodomyiini	*Orthopodomyia*	-	38	Afrotropical, Nearctic, Neotropical, Oriental, Palearctic	
Sabethini	*Isostomyia*	-	4	Neotropical	
	*Johnbelkinia*	-	3	Neotropical	
	*Kimia*	-	5	Oriental	
	*Limatus*	-	8	Neotropical	
	*Malaya*	-	12	Afrotropical, Australasian, Oriental	
	*Maorigoeldia*	-	1	New Zealand	
	*Onirion*	-	7	Neotropical	
	*Runchomyia*	2	7	Neotropical	
	*Sabethes*	5	38	Neotropical	
	*Shannoniana*	-	3	Neotropical	
	*Topomyia*	2	54	Principally Oriental	
	*Trichoprosopon*	-	13	Neotropical	
	*Tripteroides*	5	122	Principally Australasian, Oriental	
	*Wyeomyia*	15	140	Principally neotropical	
Toxorhynchitini	*Toxorhynchites*	4	88	Afrotropical, Australasian, Neotropical, Palearctic oriental, Oriental	
Uranotaeniini	*Uranotaenia*	2	265	Afrotropical, Australasian, Oriental, Neotropical	

*Modified from (1)*.

Although several environmental factors affect the geographical distribution of mosquitoes, the main ones are temperature, humidity, rains, and solar radiation. As a result of global warming, the distribution of some mosquito species has already changed, and they found ways to move toward other geographical areas. This behavior apply for mosquitoes and other insects as more tropical species have invaded temperate habitats, and temperate species have disappeared when their natural habitats have become warmer (24, 25). Anthropic intervention such as urbanization and transportation also plays an important role (26). For instance, *Ae. aegypti* originated in the forest areas of sub-Saharan Africa as a “wild,” black-pigmented insect biting species *Ae. aegypti formosus*. Facilitated by human transportation and environmental conditions a new sub-species, *Aedes aegypti (Ae. aegypti)*, evolved (27, 28) and is present in North, Central and South America, Africa, Asia and Oceania (29). It is very abundant throughout tropical and subtropical regions of America, Africa, and Asia, as well as in the Indian Ocean islands, and northern Australia (30).

### Aedes spp.

*Ae. aegypti* and *Ae. albopictus* are the most important species within this genus. Other Aedes species such as *Ae. vexans* (31), are tightly associated to allergic sensitization to mosquito bites. *Ae. aegypti* and *Ae. vexans* usually share their geographical distribution and are present almost worldwide. *Ae. aegypti* is arguably the most studied mosquito species as an allergenic source. Four salivary and six non-salivary allergens from this species have been deposited in the WHO/IUIS Allergen Nomenclature Sub-Committee (http://www.allergen.org). *Ae. aegypti* is rapidly expanding its geographical distribution and is highly concentrated in the tropics and subtropics (29) and have developed a preference for biting humans (32, 33), probably by an evolutionary over-expression of odorant receptors (34). Frequency of sensitization to *Ae. aegypti* varies depending on the region and the nature of the preparation used for diagnosis. Saliva-based preparations are probably more reliable to identify patients allergic to mosquito bites but might not be useful when sensitization occurs to non-salivary allergens. In a cohort of 34 allergic patients residing in the tropical island of Martinique, a prevalence of 65% of IgE reactivity to whole body *Ae. aegypti* extract was found (21). In Monterrey, Mexico, the frequency of IgE sensitization to *Ae. aegypti* was reported in 17.6% (5), similar to mosquito sensitization in a ~18 years old allergic population from Guangzhou, China (7). *Ae. albopictus* has become a new threat to human health as it is getting spread to new tropical, sub-tropical and temperate areas (18, 35) where it is an epidemic driver of certain diseases (36). Only two allergens from *Ae. albopictus*, Aed al 2, and Aed al 3, are in the allergen database and reports of frequency of sensitization is scarce or non-existing.

### Culex quinquefasciatus

Together with Aedes, species from Culex genera are above all other species as allergen sources. *C. quinquefasciatus* is a peridomestic insect that lives relatively farer from humans than *Ae. aegypti*. Native from west Africa, it feeds from birds, mammalians, and humans (37) and has spread out worldwide by commercial sailing, to warmer and temperate tropical and sub-tropical regions (38). At least 8 IgE reactive proteins have been detected in the saliva and 15 in whole body extracts from *C. quinquefasciatus* (15, 31) but only two allergens from this species, Cul q 2 and Cul q 3, have been reported in the databases (19). Epidemiologic data about allergy to *C. quinquefasciatus* is scarce. Seven out of 14 (50%) individuals from United States, Canada, Germany, Japan, and Switzerland who experienced systemic allergic reactions to mosquito bites were sensitized to this species (10). The high number of potential allergens found in whole body extracts of *C. quinquefasciatus* indicates that the role that this species may have in mosquito bite allergy or other clinical manifestations of allergy deserves to be studied.

An increase in the frequency of allergic sensitization to mosquitoes is expected to occur as a result of the environmental changes that have led to a global spreading of these insects. Temperature, relative humidity, and precipitations are the main factors that affect mosquito development, reproduction, and mortality. Temperature and relative humidity positively affect some mosquito species (39). High precipitations increase their population by maintaining their breeding (40). Allergies induced by mosquitoes and vector-borne diseases will become bigger threats for public health. The study of the pathophysiology and worsening of mosquito allergy will help to properly counteract the potential complications that will arise as a result of the increasing exposure to them.

## Characterized Mosquito Allergens

Mosquito allergens are divided in two main groups: (a) salivary allergens (10) and (b) body-derived allergens (8). Exposure to allergens from either group results in different clinical manifestations of mosquito allergy. Salivary allergens are mainly related to cutaneous symptoms caused by mosquito bites. We hypothesized that body allergens induce respiratory allergic symptoms after inhalation of mosquito detritus (9, 16).

### Saliva Allergens

Identification of salivary allergens is a difficult task and usually requires the extraction of saliva from the live mosquito or postmortem excision of the salivary gland which is used as the raw material to prepare allergenic extracts (41). Both methods are experimentally difficult (13, 41, 42) and result in low protein content. As an alternative, whole-body mosquito extracts could be used but salivary allergens are poorly represented in such preparations.

About 16 IgE-reactive bands (16-95 kDa) were detected by immunoblotting when saliva and salivary gland extracts from 10 different worldwide distributed mosquito species were analyzed (31). Sera from mosquito allergic individuals have specific IgE against 35.5, 32.5, and 22.5 kDa proteins present in the saliva of *C. quinquefasciatus* (42), and 14 proteins in salivary glands of *Aedes togoi, Culex tritaeniorhynchus*, and *C. pipiens pallens* with molecular weights ranging from 23 to 93 kDa (13). Some of these proteins induced an IgG1 response when used as recombinant molecules to immunize mice.

Some salivary allergens have been further characterized comprising groups 1-4 (Table 2). Usually, they needed to be produced as recombinant proteins because obtaining the natural version is a difficult task.

**Table 2:** Reported mosquito allergens.

**Allergen**	**Biological function**	**Produced as recombinant**	**Frequency of reactivity (% positives)**		Species with homolog proteins/cross-reactive allergens^*^
			**IgE**	**Skin prick test**	
**Salivary allergens**					
Aed a 1	Apyrase	rAed a 1	—	29-43	*Aedes albopictus*: **Aed al 1** *Tabanus yao*: **Tab y 1**
Aed a 2	Salivary D7 protein	rAed a 2	43	11	*Aedes albopictus*: **Aed al 2** *Culex quinquefasciatus*: **Cul q 2** *Anopheles darlingi*: **Ano d 2**
Aed a 3	Undefined 30 kDa salivary protein	rAed a 3	—	32	*Aedes albopictus*: **Aed al 3** *Culex quinquefasciatus*: **Cul q 3**
Aed a 4	α-glucosidase	rAed a 4	36	—	*Culex quinquefasciatus Aedes albopictus*
**Body derived allergens**					
Aed a 5	Sarcoplasmic Ca+ (EF-hand) binding protein	No	26.2	—	*Aedes albopictus Culex quinquefasciatus Anopheles stephensi Anopheles albimanus Anopheles sinensis*
Aed a 6	Porin 3	No	33.3	—	*Culex quinquefasciatus*
Aed a 7	Undefined protein	No	26.6	—	—
Aed a 8	Heat Shock cognate protein-70	rAed a 8	60	—	*Alternaria alternata*: **Alt a 3** *Aspergillus fumigatus*: **Asp f 12** *Dermatophagoides farinae:* **Der f 18** *Dermatophagoides pteronyssinus:* **Der p 28** *Malassezia sympodialis:* **Mala s 10** *Penicillium citrinum:* **Pen c 19** *Corylus avellana:* **Cor a 10** *Blomia tropicalis Vespa affinis* etc.
Aed a 10	Tropomyosin	rAed a 10.0101 rAed a 10.0201	33.3	—	*Anisakis simplex:* **Ani s 3** *Blattella germanica:* **Bla g 7** *Dermatophagoides farinae:* **Der f 10** *Dermatophagoides pteronyssinus:* **Der p 10** *Blomia tropicalis:* **Blo t 10** *Chironomus kiiensis*: **Chi k 10** *Crangon crangon*: **Cra a 1** *Exopalaemon modestus:* **Exo m 1** *Haliotis laevigata:* **Hal l 1** *Helix aspersa:* **Hel as 1** *Homarus americanus:* **Hom a 1** *Litopenaeus vannamei*: **Lit v 1** *Penaeus monodon:* **Pen m 1** *Periplaneta americana:* **Per a 7** *etc*.
Aed a 11	Lysosomal aspartic protease	No	40	—	**Aspartic proteases in** ^**#**^**:** *Aspergillus fumigatus:* **Asp f 10** *Blattella germanica:* **Bla g 2** *Periplaneta americana*: **Per a 2** *Solanum tuberosum:* **Sola t 2**

* *Allergen names are shown in bold and included only when reported in the WHO/IUIS Allergen Nomenclature Sub-Committee*.

# *Allergens reports as Aspartic proteases, not “Lysosomal aspartic protease” as in Ae. Aegypti*.

#### Group 1 Mosquito Allergens

The saliva apyrase (ATP di-phosphohydrolase) Aed a 1, from *Ae. aegypti*, is the only allergen from group 1 that has been accepted by the WHO/IUIS Allergen Nomenclature Sub-Committee. It corresponds to a 68 kDa enzyme with homology with the 5′-nucleotidase enzyme family (43) and interferes with platelet aggregation in human blood by hydrolyzing ADP and ATP released by the platelets and other cells (44). About 29% of Canadian individuals sensitized to mosquito bites had positive SPT to rAed a 1 (11). However, when tested in an allergic population from the tropics, living in urban and sub-urban areas, the IgE frequency of reactivity increased to 60% (19). B cell epitopes seem to be contained in the 150-562 amino acid region and react with the IgE and IgG from allergic individuals (45). Homolog molecules or apyrase enzymatic activity have been detected in the saliva from *Ochlerotatus triseriatus, Ochlerotatus hendersoni* (46), and *Ae. albopictus* (31, 47).

#### Group 2 Mosquito Allergens

It corresponds to allergens that belong to the family of proteins called D7, which are required by mosquitoes for feeding and reproduction, and are released together with the saliva during biting. They have structural homology with the protein THP12 from *Tenebrio molitor*, which is part of the family of pheromone-binding proteins and odorants and help transporting hydrophobic molecules (48). Allergens within this group have been reported in the WHO/IUIS allergen database from *Ae. aegypti* (49) and in *Ae. albopictus, An. dirus*, and *C. quinquefasciatus* (19). This group could also be present in other Aedes species and *O. triseriatus* (31).

Aed a 2, from *Ae. aegypti*, is a multi-domain protein with a N-terminal and a C-terminal domain that binds leukotrienes and biogenic amines released as a mechanism of protection in individuals that are getting bitten (50). In a group of 15 mosquito bite allergic individuals residing in the tropics the frequency of reactivity was 100%, studied by immunoblotting using salivary gland extracts (19). However, in a North American population seems to be 11% (31). Recombinant Aed a 2 expressed in insect cells infected with baculovirus retains the IgE-binding capacity and allergenicity, and immunogenicity as seen in immunized mice (51), suggesting that it can be used as a replacement of the natural protein.

#### Group 3 and 4 Mosquito Allergens

The WHO/IUIS allergen database reports allergens in groups 3 and 4 from the mosquito species *Ae. aegypti* (52, 53), *Ae. albopictus* and *C. quinquefasciatus* (19). Aed a 3 and Aed al 3 in *Ae. aegypti* and *Ae. albopictus*, respectively, are 30 kDa molecules. In *C. quinquefasciatus*, Cul q 3 is a 35 kDa molecule. Aed a 3 from *Ae. aegypti* shows collagen binding capacity and prevents its interaction with platelet glycoprotein IV, integrin α2β1 and von Willebrand factor (52). When used together with Aed a 1 and Aed a 2, about 60% of an allergic population could be accurately diagnosed (53). 40% of individuals from a tropical region react against Aed a 3. Aed a 4 is a 67 kDa α-glucosidase. About 36-46% of mosquito allergic individuals react against this allergen (19, 54).

### Body-Derived Allergens

Allergic individuals have IgE against non-salivary body-derived mosquito proteins. For instance, in the subtropical city of Yazd, Iran, 33% of individuals with allergic rhinitis had positive skin test to whole body mosquito extracts (55). Similar observations were reported in India where 47% of the population with asthma and/or rhinitis were sensitized to mosquito allergens (6) and in Martinique with 65% of sensitization (16). Such observations strongly suggest that exposure to mosquito allergens occurs through the skin when the mosquito is biting, but also through the airways, leading to different manifestations of the allergic response such as asthma and rhinitis.

An important question to address is whether body-derived mosquito allergens are found in the dust or mattresses from the allergic individuals' residing places and in quantities enough to induce allergic symptoms. Although we don't know the answer yet, several studies have made important advances in this matter. To begin, extracts prepared from airborne particles collected in the homes of mosquito allergic individuals block the specific IgE reactivity of sera from such individuals to whole-body *C. quinquefasciatus* extract (14), which allows to hypothesize that mosquito allergens are present in house dust and retains antibody binding capacity. A weakness of this hypothesis is that it is based on immunoassays, and it cannot exclude that arthropod-derived allergens might be the molecules responsible of inhibiting the IgE binding capacity. It is already demonstrated that they are present in the dust from places where allergic individuals reside (56, 57). The DNA-based study of arthropod diversity in homes via high-throughput marker gene sequencing of 700 home's dust revealed that mosquito (*Aedes spp*) together with carpet beetle, dust mite and Aphid (*Aphis spp*) are common in home's dust (58). Quantitative analyses are necessary to establish whether the amounts of mosquito allergens in such samples are high enough to represent a potential primary sensitizer and inducer of allergic symptoms.

Different allergen composition has been observed depending on the sample and techniques used to detect IgE binding molecules. There are at least 11 IgE-binding proteins in whole-body *Ae. aegypti* extract, as detected by immunoblotting (16). Five of those proteins cross-react with allergens from HDM, cockroach and shrimp. Whole-body extracts are prepared by extraction with PBS and non-PBS soluble allergens could be missing. The analysis of the *Ae. aegypti* allergenome using proteomic tools revealed a set of 25 IgE-binding molecules corresponding to 10 different proteins and some of their variants or isoforms (8). Four of them were deposited in the WHO/IUIS Allergen Nomenclature Sub-Committee as Aed a 5.0101 (sarcoplasmic Ca^+^ (EF-hand) binding protein), Aed a 6.0101 (Porin 3), Aed a 7.0101 (undefined protein), Aed a 8.0101 (HSC-70), and Aed a 11.0101 (lysosomal aspartic protease). Notice that tropomyosin Aed a 10 was also identified. Only the HSC-70, Aed a 8 and tropomyosin Aed a 10 have been further studied (Table 2).

#### Group 8 Mosquito Allergens

Aed a 8 is the representative allergen of this group. Heat shock cognate protein-70 belongs to the highly conserved Heat shock protein-70 family (59), chaperones that help in protein folding maintaining their correct biological function under stress conditions (60). Homolog allergens are present in *Dermatophagoides farinae* (61) and cockroach (62). Aed a 8 reacted with the IgE in 9 out of 15 allergic individuals (60%) (8). Similar frequency of reactivity is reported for Der f 8 from *D. farinae* (61).

We obtained recombinant Aed a 8 as a 74 kDa by expression in *Escherichia coli*. Recombinant Aed a 8 inhibited 43% of the IgE reactivity of a mixture of human serum samples to the whole body extract of *Ae. aegypti*, indicating that the wild type Aed a 8 is present in such extract, and retains immunogenicity and the capacity to activate basophils. Six out of 14 sera from allergic individuals reacted to the recombinant and, when used to immunize mice, it induced specific antibody that also reacted against the natural counterpart, indicating that it retained biological activity (63).

Obtaining mosquito allergens is a difficult task, especially for proteins that are expressed in low levels, such as HSC-70 molecules. Using purified and biologically active recombinant allergens will help to overcome this problem and we strongly suggest using rAed a 8 for further analysis of mosquito allergy and study the clinical relevance of group 8 allergens in the physiopathology of mosquito allergy.

#### Group 10 Mosquito Allergens

Tropomyosin is a well-described allergen from diverse sources. Some of the allergenic sources are shrimps, lobsters, prawns, crabs, fish, mollusks, and snails. This allergen is also common in HDMs, helminths, cockroaches, and insects, and partially explains the existence of the cross-reactivity between them (64, 65). *Ae. aegypti* has 11 genes that encode different variants, or isoforms of tropomyosin. Four of them were detected, characterized and purified (66). Two tropomyosin isoforms, Aed a 10.0101 and Aed a 10.0201 are the most abundant and 33% of a population sensitized to *Ae. aegypti* had IgE against a mixture of them (66), suggesting that they are relevant molecules involved in IgE sensitization against *Ae. aegypti* tropomyosins.

The IgE frequency of sensitization to tropomyosin is variable, but usually low. Tropomyosin from shrimp species *Penaus aztecus*, Pen a 1, binds up to 75% of shrimp-specific IgE antibodies (67, 68). In Africa and South America, the prevalence of sensitization to mite tropomyosin is ~50% (69, 70), higher than that in developed countries (71, 72). The relatively high frequency of sensitization to tropomyosin in African and South American areas indicates that cross-reactivity with mosquito tropomyosin must be considered.

## IgE Cross-Reactivity Mediated by Mosquito Allergens

**Figure 1:**
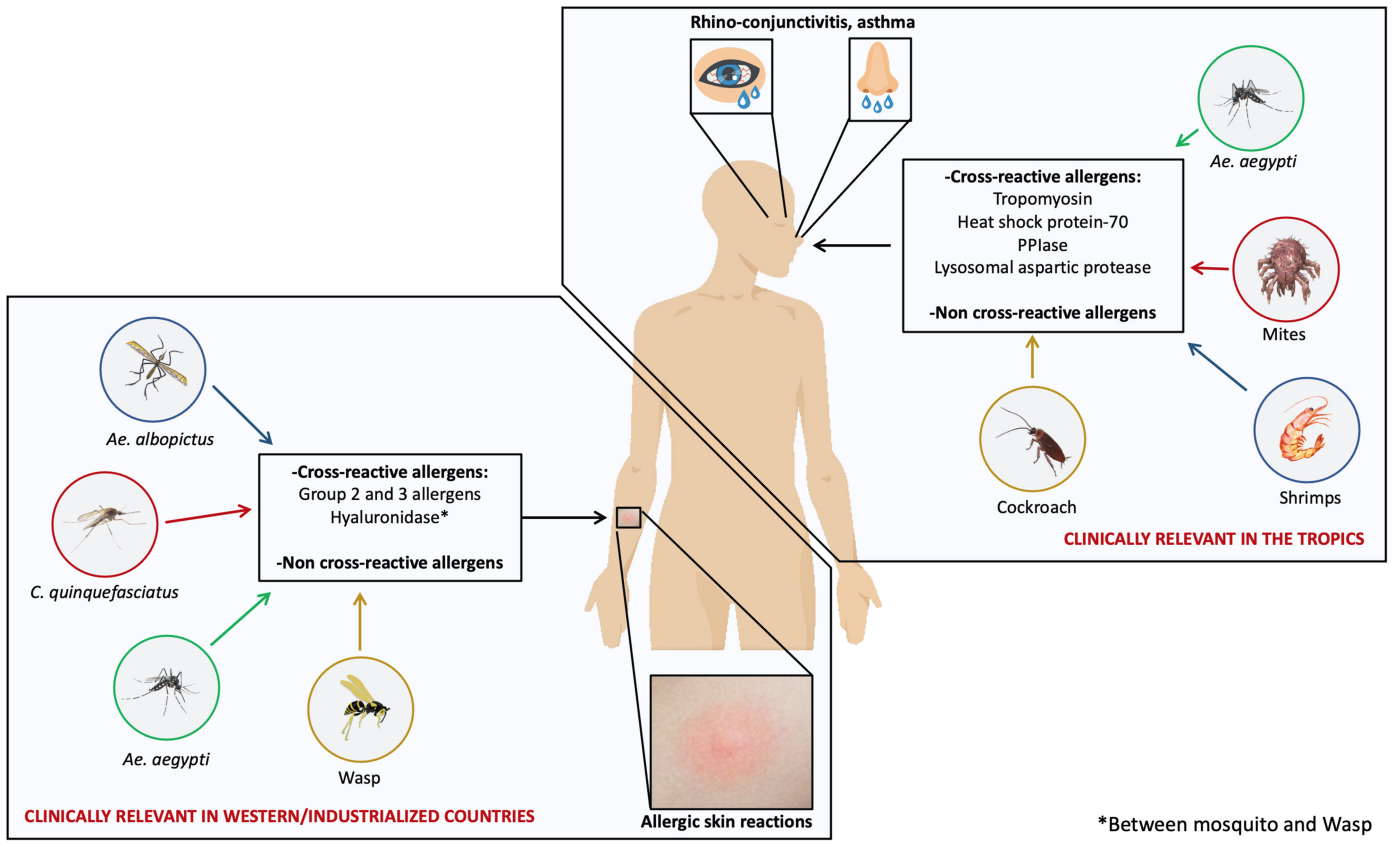
Unique and cross-reactive mosquito allergens induce different manifestations of mosquito allergy. Mosquito species such as *Ae. aegypti, Ae. albopictus*, and *C. quinquefasciatus* are distributed worldwide; however, some studies suggest that two kinds of allergic reactions induced by mosquito allergens are clinically relevant depending on the geospatial location: allergic skin reactions induced by salivary allergens and respiratory reactions induced by body-derived allergens. Skin reactions are common in western and industrialized countries and respiratory reactions are relevant in tropical areas. Cross-reactivity between mosquito species and with several species within Arthropods may play an important clinical role.

The apparent geospatial differences of immune and allergic response to mosquito allergens have implications in the cross-reactivity phenomena. In regions where cutaneous allergic reactions to mosquito bites is frequent, saliva-derived allergens are the main cross-reactive molecules (15, 31, 73, 74). In contrast, in tropical areas, body allergens seem to be the main proteins associated to cross-reactivity with arthropods (8, 16) (Figure 1). These differences have clinical implications since preparations for diagnostic and immunotherapy based on salivary allergens would make sense to consider in western and industrialized countries. The case is different for tropical and subtropical countries where species specific and cross-reactive body-derived allergens might be the best targets to focus on. It is also possible that in these regions, body-based preparations could be a more effective tool to cope with allergies caused by mosquitoes and other arthropods.

### Cross-Reactivity Mediated by Saliva Allergens

Studies on animals indicate that sensitization to a mosquito salivary allergen induce antibodies that react against allergens from different mosquito species. Sera from rabbits immunized with rAed a 1 cross-react with extracts from *Ae. vexans* and *Ae. albopictus* (31). The finding of homologs of the apyrase Aed a 1 allergen in *Ae. aegypti, O. triseriatus*, and *O. hendersoni* indicates that this protein is conserved among several mosquito species and explains the above-mentioned observations. Similarly, immunization with rAed a 2 induces anti-sera that react with extracts of *C. quinquefasciatus, O. triseriatus* (46) and several species of *Aedes* (12, 31). It is plausible to assume that saliva proteins other than group 1 and 2 allergens are involved in the cross-reactivity among mosquito species.

Several studies show a similar phenomenon in humans. Individuals from Shanghai, China, have IgE-reactivity to *Ae. vexans* allergens, although this species is not indigenous in such area (31, 73). Contrarily, *Ae. vexans* is a major pest in Winnipeg, Manitoba (Canada) where individuals allergic to mosquitoes co-react with allergens from other mosquito species not found in Manitoba (73). The sera from individuals allergic to mosquito bites in Thailand react with several broad range molecular weight proteins present in the extracts from the *C. quinquefasciatus, Ae. aegypti, Ae. albopictus*, and *An. minimus*, common mosquitoes (15).

Saliva derived allergens from mosquitoes can also cross-react with proteins from wasps. The so-called “wasp/ mosquito syndrome,” involves an IgE cross-reactive 44-kDa hyaluronidase which is present in both insects (74). Cross-reactivity between salivary allergens occurs in western/industrialized countries as well as in tropical regions. However, it is necessary to evaluate the clinical implications that this may have. In countries like Canada where cross-reactivity among *Ae. vexans* and several other mosquito species is common (31, 73) and mosquito bite allergies are frequent, it is important to determine whether such cross-reactivity has implications in the physiopathology of allergic responses. However, in other regions like Brazil, cross-reactivity between endemic mosquito species also occur (48), but it involves antibodies from allergic and non-allergic individuals. This suggests that in such regions, broad sensitization to mosquito occurs but does not mean that it leads to a clinical manifestation of allergy and cross-reactivity might not be important.

### Cross-Reactivity Mediated by Body-Derived Allergens

There are homolog proteins distributed in several species from the filum Arthropoda, including mosquitoes, that induce allergic reactions. It is widely accepted that in the tropics HDMs, cockroaches and shrimp are some of the most common sources of allergens (75).

*in vitro* studies and SPTs showing that individuals sensitized to one or several arthropod species had concomitant immunoreactivity against mosquito proteins or extracts led to the hypothesis that cross-reactivity involving allergens from mosquitoes and other sources occurs (76, 77) (Figure 1).

In our mentioned study with allergic individuals from Martinique (16), we identified four novel cross-reactive allergens in *Ae. aegypti* allergen extract and concluded that, these molecules could influence the manifestation of allergy to environmental allergens in the tropics. ELISA experiments showed that in this population *D. pteronyssinus, Litopenaeus vannamei, Blomia tropicalis*, and *Periplaneta americana* extracts inhibited the IgE reactivity to *Ae. aegypti* extract in 75.4-96.6%, and that the main allergen involved was tropomyosin (16), a well-known cross-reactive molecule within arthropods. Besides tropomyosin, other components are involved, especially a 17.9 kDa PPIase that has 81.1% identity in the amino acid sequence with Der f 29 allergen from *D. farinae*.

Tropomyosin is the main cross-reactivity allergen in *Ae. aegypti*, which is expressed as several variants and isoforms. Two of the more abundant are Aed a 10.0101 and Aed a 10.0201, which cross-react with rDer p 10 from *D. pteronyssinus* (78). In the Caribbean, 33% of a group of sera from allergic individuals had specific IgE to these two tropomyosins (9); a number that is evidently higher than the frequency of sensitization to tropomyosins from other sources typically observed in developed countries.

We demonstrated that cross-reactivity of *Ae. aegypti* tropomyosins leads to effector cell activation. We used basophils in the PBMCs from non-allergic donors where the membrane bound IgE was stripped away and re-sensitization with sera from allergic patients sensitized to the tropomyosin Der p 10. Challenging such cells with rDer p 10 or recombinant *Ae. aegypti* tropomyosins, induced dose dependent activation. In addition, splenocytes from mosquito tropomyosin immunized mice proliferate upon stimulus with rDer p 10 (78).

## Diagnosis

Whole body extracts prepared from *Ae. communis, C. pipiens* or *C. quinquefasciatus* are currently the main preparations used for the purpose of mosquito allergy diagnose, although their use has some disadvantages. To begin, the accuracy of the diagnosis is compromised when the primary sensitizer is a species different to the one used to prepare the allergenic extract. Different geographical regions have different local mosquito species and having the appropriate mosquito extract that works for a specific population, is mandatory to achieve an appropriate diagnosis (19), but sometimes is not possible. For instance, *Ae. communis* is endemic in northern temperate zones but poorly present in tropical countries where *Ae. aegypti* and *C. quinquefasciatus* are abundant (18). The use of *Ae. communis* extract results in poor diagnosis of mosquito allergic individuals from the tropics (19, 79). In contrast to the case in Cuba, where mosquito allergy is frequently related to *C. quinquefasciatus* bites and using a high dose of standardize extract of this mosquito species in SPTs resulted in positive results that correlated in 100% of the patients (80). Second, whole body extracts may have poor representation of saliva allergens (15, 81), which could jeopardize the accuracy of such preparations to detect allergic individuals who are sensitized to the saliva (79). Wang et al. found that the diagnosis by the detection of specific IgE using salivary extracts provide higher specificity and sensitivity than using whole body extracts (82). Alternatively, using saliva-based preparations or salivary gland extracts, may provide 80% positivity result (4). However, this is not cost effective and requires complicated procedures that result in low recovery of allergens. Using whole-body extracts appear more attractive when the affected population is sensitized to non-salivary allergens.

Using recombinant allergens is especially convenient to circumvent the above mentioned problems as they are obtained in high amounts and purity. Additionally, they have the intrinsic advantages when used as a replacement of natural extracts, as they can be easily standardized, subjected to proper quality control analysis and allows component-resolved immunotherapy since it help to identify the set of allergens to which each individual is sensitized (83–85). Only a few recombinant mosquito allergens have been obtained and analyzed. Aed a 1, Aed a 2, and Aed a 3 have been well-characterized, obtained as recombinants and are an interesting tool to replace *Ae. aegypti* saliva since a mixture of the three allergens allows identifying 60% of the *Ae. aegypti* population allergic to mosquito bites (53). Evidently, clinically relevant mosquito allergens must be chosen to allow a better identification of allergic individuals (86). Obtaining recombinant saliva allergens from other species is also necessary to allow future development of more accurate diagnostic tests.

The situation is similar for individuals sensitized to non-salivary allergens. Very few body allergens have been detected and only two recombinant allergens from *Ae. aegypti*, rAed a 8, and rAed a 10 (9), have been produced and tested. We made some advances and proposed an alternative to replace whole body *Ae. aegypti* extracts for a mixture of three allergens, Aed a 6, Aed a 8, and Aed a 10, which may be enough to identify more than 80% of the allergic individuals (8). More efforts must be done to broadly identify and characterize saliva and body mosquito allergens from different species, obtain relevant allergens as recombinant proteins and confirm their potential as diagnostic tools in clinical studies with well-characterized populations.

## Concluding Remarks

The concept of mosquito allergy should be re-evaluated as more allergens have been identified, revealing that they belong to the saliva and the insect's body. Mosquito body allergens seem to induce different types of allergic responses, such as asthma, allergic rhinitis, and probably conjunctivitis. The mechanisms of exposure to these allergens are not established yet but, may occur by inhalation of mosquito detritus suspended in the air. These observations have several implications and open many questionings: (1) Is there a relationship between the exposure to mosquito allergens and the onset of respiratory allergic reactions? (2) Do mosquito allergens induce manifestation of allergic responses different to the cutaneous or airway related symptoms? (3) Could mosquito allergens contained in the environment induce immunological responses?

The current knowledge has many unresolved issues. Only a few allergens have been identified and characterized, and they belong to a few species, mainly *Ae. aegypti* and *C. quinquefasciatus*. The diversity of mosquito species is quiet variable depending on the geographical region and it has continuously changed with global warming. Additionally, an important degree of cross-reactivity occurs among mosquitoes and several arthropod species. The effects that this phenomenon has on the pathophysiology of allergy diseases is still unknown.

The quest for answers to these questions will help to propose a more accurate definition of mosquito allergy and may pave the way to find solutions to the scientific and clinical challenges that will subsequently arise. More efforts must be done to identify and characterize saliva and mosquito body allergens from different species, obtain relevant allergens as recombinant proteins and confirm their potential as diagnostic tools in clinical studies with well-characterized populations.

## Author Contributions

LP conceived the idea. LP and JC contributed equally to the preparation of the draft and final manuscript. All authors contributed to the article and approved the submitted version.

## Conflict of Interest

JC was employed by the company Inmunotek, S.L. The remaining author declares that the research was conducted in the absence of any commercial or financial relationships that could be construed as a potential conflict of interest.
